# Mechanistic Exploration of Aristolochic Acid I-Induced Hepatocellular Carcinoma: Insights from Network Toxicology, Machine Learning, Molecular Docking, and Molecular Dynamics Simulation

**DOI:** 10.3390/toxins17080390

**Published:** 2025-08-05

**Authors:** Tiantaixi Tu, Tongtong Zheng, Hangqi Lin, Peifeng Cheng, Ye Yang, Bolin Liu, Xinwang Ying, Qingfeng Xie

**Affiliations:** 1 Department of Physical Medicine and Rehabilitation, The Second Affiliated Hospital and Yuying Children’s Hospital of Wenzhou Medical University, Wenzhou 325035, China; tutiantaixi@wmu.edu.cn (T.T.);; 2 Renji College, Wenzhou Medical University, Wenzhou 325035, China; 3 The First School of Medicine, Wenzhou Medical University, Wenzhou 325035, China; 4 Chengdu Bayi Orthopedic Hospital, Chengdu 610031, China

**Keywords:** aristolochic acid I, hepatocellular carcinoma, network toxicology, machine learning, molecular docking

## Abstract

This study explores how aristolochic acid I (AAI) drives hepatocellular carcinoma (HCC). We first employ network toxicology and machine learning to map the key molecular target genes. Next, our research utilizes molecular docking to evaluate how AAI binds to these targets, and finally confirms the stability and dynamics of the resulting complexes through molecular dynamics simulations. We identified 193 overlapping target genes between AAI and HCC through databases such as PubChem, OMIM, and ChEMBL. Machine learning algorithms (SVM-RFE, random forest, and LASSO regression) were employed to screen 11 core genes. LASSO serves as a rapid dimension-reduction tool, SVM-RFE recursively eliminates the features with the smallest weights, and Random Forest achieves ensemble learning through decision trees. Protein–protein interaction networks were constructed using Cytoscape 3.9.1, and key genes were validated through GO and KEGG enrichment analyses, an immune infiltration analysis, a drug sensitivity analysis, and a survival analysis. Molecular-docking experiments showed that AAI binds to each of the core targets with a binding affinity stronger than −5 kcal mol^−1^, and subsequent molecular dynamics simulations verified that these complexes remain stable over time. This study determined the potential molecular mechanisms underlying AAI-induced HCC and identified key genes (CYP1A2, ESR1, and AURKA) as potential therapeutic targets, providing valuable insights for developing targeted strategies to mitigate the health risks associated with AAI exposure.

## 1. Introduction

AAI is a nitrophenanthrene carboxylic acid compound derived from plants of the Aristolochia genus and is a key bioactive chemical compound responsible for the biological functions exhibited by these plants [1]. Historically, dozens of herbs containing aristolochic acids (AAs) and related drugs have been used in traditional Chinese medicine (TCM), which comprises complementary and alternative medicines, to treat diseases such as eczema, inflammation, stroke, respiratory infections, gout, coronary artery disease, hypertension, heart failure, and snakebites [2]. However, the health impacts of AAI are a double-edged sword [3]. In recent years, numerous studies have demonstrated that AAI has potent genotoxic and carcinogenic properties, and that its long-term intake is a major pathogenic factor in diseases such as aristolochic acid nephropathy (AAN) and HCC [4,5]. AAI primarily poses health risks due to the intake of traditional herbal medicines, but its characteristics as a persistent environmental contaminant have gained widespread attention in recent years [6]. A study has shown that AAI has high chemical stability in soil and water, and can enter the human body through groundwater contamination, contaminated groundwater, wheat crops, and flour in Balkan countries [7]. Additionally, this long-term low-dose exposure pattern may play an important role in regions with high HCC incidence. Unfortunately, the specific carcinogenic molecular mechanisms remain insufficiently explored. Although there are significant variations in HCC incidence patterns, studies in Taiwan and East Asia have revealed that AAI-associated characteristic mutations (single-base substitution mutational signature 22, SBS22) account for as many as 78% of HCC patients [8,9]. As AAI is one of the leading causes of cancer-related mortality worldwide, studying the specific mechanisms by which AAI induces HCC under environmental exposure conditions is highly important for public health [10,11].

In the 1990s, Vanherweghem et al. first reported that more than 100 young women in Belgium who used weight-loss drugs containing Fangchi (a plant containing AAI), suffered kidney damage and required kidney transplants [12]. Some patients later developed kidney cancer and bladder cancer. Kidney damage caused by AA was subsequently confirmed and identified as AAN in European countries, Asia, the United States, and worldwide. Some studies have also indicated an increased risk of bladder cancer and upper tract urothelial carcinoma in AAN patients, which has attracted widespread attention worldwide [13,14]. The International Agency for Research on Cancer classified AAI as a human carcinogen (Group I) because of its carcinogenic, mutagenic, and genotoxic mechanisms [15].

Several years later, it was discovered that aristolochic acid is closely associated with the mechanisms of liver cancer development [16]. In 2013, Poon et al. first reported the AA mutation signature SBS22 characterized by A: T-to-T: A changes in liver cancer cells [17]. Another study revealed 10 cases of SBS22 in the genomic data of 88 Chinese liver cancer patients, supporting the pathogenic role of AA in liver cancer [10]. Ng et al. further elaborated on those findings in 2017 and considered toxic herbs containing AA as a significant cause of liver cancer in Asia. Their study revealed that 76 out of 98 patients (78%) in Taiwan had characteristic AA-related mutations. They also examined 1400 HCC samples from China, Japan, Korea, Southeast Asia, and North America, where the prevalence of SBS22 reached as high as 56% [8]. In the same year, relevant studies linked aristolochic acid and its derivatives to liver cancer in Taiwan and various parts of Asia, establishing a decisive association and bringing aristolochic acid back into the forefront of research [18]. These data suggest that AA signatures are closely related to HCCs in Asia. Since then, AA has been widely discussed and quickly became a focal point of public debate. AAI, as a persistent soil contaminant, is widely present in various environmental media such as soil and water; it is also a component of endemic weeds in wheat fields [19]. Some of the most significant and well-documented routes of exposure include wheat flour contaminated with aristolochic seeds and traditional medicine using various formulations of aristolochic plants [19]. Other indirect sources of contamination include soil, groundwater, and cultivated farm plants. All studies detecting low levels of AAI support the idea that prolonged exposure to this biological contaminant, which behaves like a persistent organic pollutant in the environment, may lead to bioaccumulation in humans. Previous studies have confirmed that AAI has DNA mutation, gene damage, and cellular oxidative stress properties, and can induce gene mutations through the formation of DNA adducts [18]. Especially in East Asia, the high prevalence of AAI-specific SBS22 mutations in HCC patients offers compelling molecular evidence of its carcinogenicity, primarily linked to TCM use, while also hinting at potential environmental exposure risks. In conclusion, the current research has not adequately addressed critical gaps in the understanding of the molecular mechanisms of AAI-induced HCC, particularly in East Asia, where herbal medicine use remains the primary cause of AAI-related hepatocarcinogenesis. While environmental exposure may represent an emerging risk factor for HCC, further studies are needed to confirm this trend and elucidate its impact on the immune microenvironment.

HCC is a complex disease, characterized by the uncontrolled growth of liver cells, and is influenced by genetic mutations, hormonal changes, and environmental factors [20]. As the liver plays a critical role in detoxification and metabolism, it is particularly vulnerable to carcinogens such as AAI. The damage caused by AAI to the liver and its carcinogenic effects have garnered increasing attention. The genotoxicity of AAI leads to the formation of DNA adducts, a key factor in its carcinogenic potential [21]. Moreover, as a persistent environmental toxicant, AAI is a significant contributor to the development of liver cancer. Not only does it exacerbate genetic susceptibility, but it may also promote the occurrence of liver diseases such as cirrhosis, which can ultimately progress to hepatocellular carcinoma [6,22].

Although studies have shown that AAI has strong carcinogenic properties, its specific molecular toxicity mechanisms have not yet been fully elucidated. Therefore, further research on how AAI interacts with cellular signaling pathways to promote cancer development and other related health issues is a key focus of current investigations [17]. Network toxicology provides an interdisciplinary approach that combines systems biology and toxicology to investigate in depth how chemicals interact with biological systems, enabling a more accurate description and prediction of the toxicological properties of drugs [12]; it offers a new perspective for elucidating the molecular mechanisms of the adverse effects of traditional Chinese medicine compounds [23]. This paper comprehensively reviews toxicity prediction tools and common databases [24] and constructs molecular networks using the STRING database to identify key nodes and signaling pathways involved in AAI-induced toxicity [25]; it also examines the focus of network toxicology research in the context of safety evaluations of traditional Chinese medicines [16,26].

To address the existing challenges and limitations of network toxicology, we employed three mainstream machine learning algorithms: support vector machine recursive feature elimination (SVM-RFE), random forest, and LASSO regression. SVM-RFE enhances model performance by iteratively removing less important features [27], whereas random forest improves accuracy and robustness by constructing multiple decision trees and providing feature importance rankings [28]. LASSO regression performs variable selection through L1 regularization, making it particularly effective for high-dimensional datasets. Finally, SHAP analysis was conducted to further evaluate the importance of each variable in the machine learning process. Molecular docking is a computational simulation technique used to predict the binding modes and affinities between ligands and receptors [29]; it is used not only in computer-aided drug design but also to study the interactions and affinities between compounds and target genes [30]. Molecular dynamics simulations dynamically display the binding stability between small molecules and proteins, further elucidating their potential toxic mechanisms and assisting in the identification of new intervention targets [31].

This study pursues a step-wise strategy to clarify how AAI triggers hepatocellular carcinoma. First, network toxicology and machine learning pinpoint the most relevant molecular targets. Next, the predicted interactions are examined in detail through molecular docking. Then, molecular dynamics simulations validate the structural stability of the AAI–protein complexes. Finally, our study is validated using immunohistochemistry (IHC) from an HPA database [32]. We explored potential binding sites and constructed a comprehensive AAI carcinogenic model, while also exploring potential targeted intervention strategies. This research promotes the application of bioinformatics in the study of pathogenic mechanisms induced by environmental pollutants [33].

## 2. Results

### 2.1. Toxicity Prediction for AAI

**Figure 1:**
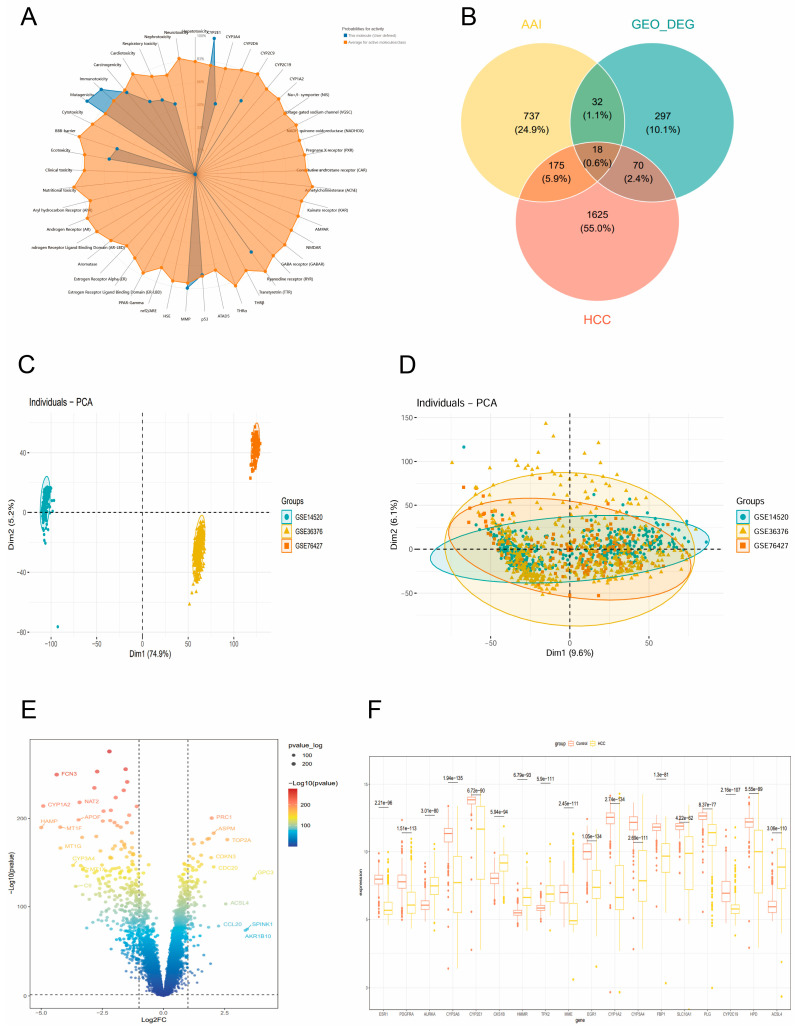
Identification of core toxicity-related targets. (**A**) Toxicity radar chart depicting the multi-dimensional toxicity profile. (**B**) Venn diagram after the intersection of the AAI, HCC target, and GEO database difference analysis results. (**C**) Principal Component Analysis (PCA) plot before batch effect correction. (**D**) PCA plot after batch effect removal, demonstrating improved dataset integration. (**E**) Volcano plot of DEGs from GEO datasets, highlighting significantly dysregulated genes. (**F**) Multi-group boxplot illustrating the expression patterns of core toxicity-related targets.

Using the ADMET and ProTox-II databases, we predicted the toxicity profile of AAI (aristolochic acid I) and identified its significant potential for carcinogenic mutagenicity and hepatotoxicity. The analysis revealed that AAI exhibits a high likelihood of inducing carcinogenic mutations, aligning with its well-documented role in promoting tumorigenesis. Additionally, AAI was predicted to have a pronounced impact on liver injury, further supporting its association with HCC and other liver-related pathologies. These findings underscore the dual toxicological effects of AAI, highlighting its carcinogenic and hepatotoxic properties, which may contribute to its role as a potent environmental carcinogen and liver-damaging agent. This predictive analysis provides critical insights into the mechanisms underlying AAI-induced toxicity and its potential implications for human health (Figure 1A).

### 2.2. Search of Targets for AAI and HCC

In our analysis, we identified a total of 962 targets associated with AAI and 1888 targets related to HCC. Through intersection analysis, we found 193 overlapping targets shared between AAI and HCC. These common targets represent potential key molecular players that may underlie the mechanisms by which AAI contributes to the development or progression of HCC. The identification of these shared targets provides a foundation for further investigation into the molecular pathways linking environmental exposure to AAI and the pathogenesis of HCC, offering valuable insights for future therapeutic and preventive strategies.

### 2.3. Recognition Core Gene from GEO

Through a differential expression analysis of the GEO dataset, we identified a total of 417 differentially expressed genes (DEGs), which were subsequently narrowed down to 18 key intersection genes. Volcano plots were utilized to visualize the expression patterns of these genes, revealing that genes such as GPC3 and SPINK1 were significantly upregulated in the tumor group, while genes including HAMP and CYP1A2 were markedly downregulated. These findings highlight the distinct transcriptional changes associated with tumorigenesis and provide insights into potential molecular drivers of the disease.

Furthermore, multi-group boxplots were employed to compare gene expression levels between tumor and normal groups. Among the analyzed genes, CYP1A2 exhibited the most significant differential expression, with its expression levels being notably lower in the tumor group compared to the normal group. This pronounced difference underscores the potential role of CYP1A2 in tumor biology and suggests its importance as a candidate biomarker or therapeutic target.

Overall, these results demonstrate the utility of differential expression analysis in uncovering key genes associated with tumor development. The integration of visualization tools, such as volcano plots and boxplots, provides a clear and comprehensive representation of the transcriptional landscape, emphasizing the critical involvement of genes like CYP1A2 in the molecular mechanisms underlying the disease (Figure 1B–F).

### 2.4. Genetic Screening Based on Machine Learning

**Figure 2:**
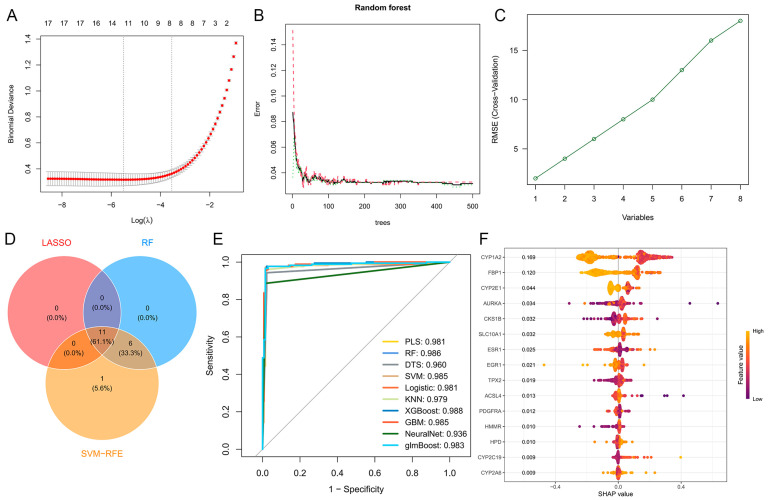
Machine learning-based identification and validation of key targets. (**A**–**C**) Feature selection using LASSO regression, Random Forest, and Support Vector Machine algorithms. (**D**) Venn diagram of consensus targets identified by all three machine learning methods. (**E**) ROC curves evaluating the predictive performance of the integrated model. (**F**) SHAP analysis for interpretability of model predictions.

Using three distinct machine learning algorithms, we systematically screened 18 candidate genes and identified 11 key target genes that exhibited significant relevance in the context of our network toxicology analysis. To validate the robustness and predictive performance of these genes, we employed 10 machine learning algorithms and evaluated their performance using receiver operating characteristic (ROC) curves. The area under the curve (AUC) values for all models exceeded 0.96, demonstrating exceptional accuracy and reliability in distinguishing relevant biological patterns (Figure 2A–E).

Furthermore, SHapley Additive exPlanations (SHAP) analysis was conducted to interpret the contribution of each gene to the machine learning models. This analysis highlighted the critical importance of CYP1A2, AURKA, and ESR1, as these genes consistently ranked among the top contributors across multiple models. Their significant SHAP values underscore their pivotal roles in the predictive performance of the algorithms and suggest their potential as central players in the molecular mechanisms under investigation (Figure 2F).

These findings not only validate the robustness of our machine learning approach but also emphasize the biological significance of CYP1A2, AURKA, and ESR1 in the context of network toxicology. The integration of multiple machine learning algorithms and interpretability tools provides a comprehensive and reliable framework for identifying and prioritizing key genes in complex biological systems.

### 2.5. Construction of Protein–Protein Interaction Network and Hub Targets Screening

**Figure 3:**
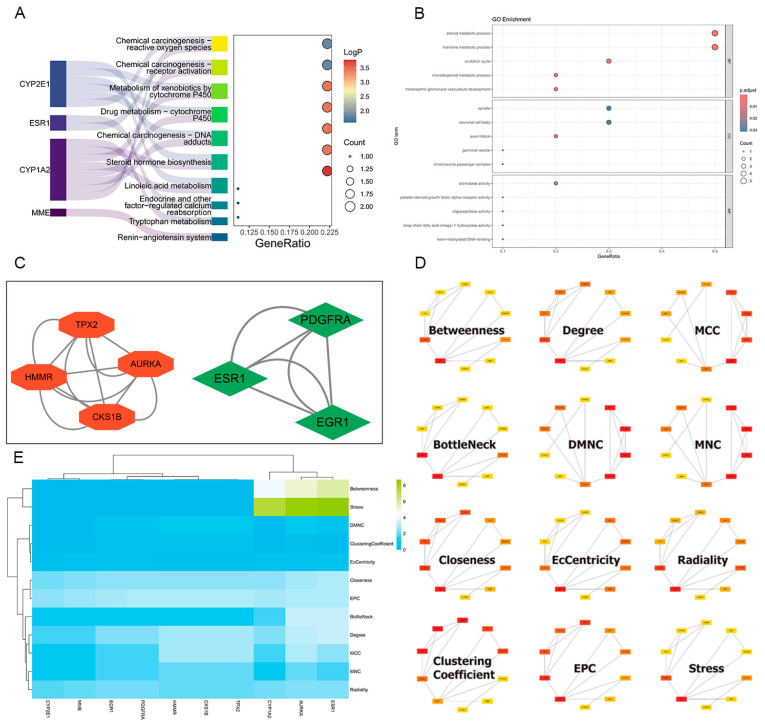
Functional enrichment and network analysis of core targets. (**A**) KEGG pathway enrichment analysis of key toxicity-associated pathways. (**B**) Gene Ontology (GO) enrichment analysis covering biological processes, molecular functions, and cellular components. (**C**) PPI network clustering via the MCODE algorithm. (**D**) Hub gene identification using 12 topological algorithms. (**E**) Visualization of hub genes and their interactions within the PPI network.

Using Cytoscape’s cytoHubba algorithm, we comprehensively analyzed the protein–protein interaction (PPI) network and identified CYP1A2, ESR1, and AURKA as key hub genes across multiple topological levels. These genes exhibited significant centrality measures, including degree, betweenness, and closeness, underscoring their pivotal roles in the network. The results are visualized as a heatmap, which highlights the relative importance of these hub genes in maintaining the network’s integrity and functionality (Figure 3C,D).

Additionally, the MCODE algorithm was employed to detect densely connected network modules, revealing that AURKA and ESR1 play critical roles in two distinct modules. These modules are likely to represent functional clusters involved in specific biological processes or pathways, further emphasizing the importance of AURKA and ESR1 in the network.

Together, these findings demonstrate that CYP1A2, ESR1, and AURKA serve as central hubs within the PPI network, potentially orchestrating critical cellular processes and signaling pathways. Their identification as key nodes provides valuable insights into the molecular mechanisms underlying the network’s dynamics and highlights their potential as therapeutic targets in the context of network toxicology (Figure 3E).

### 2.6. GO and KEGG Enrichment Analysis

In the MF category, genes were enriched in activities such as platelet-derived growth factor alpha-receptor activity and fructose 1,6-bisphosphate 1-phosphatase activity. The BP category revealed significant enrichment in processes like hormone metabolic process and steroid metabolic process. For CC, genes were associated with components like the chromosome passenger complex and germinal vesicle. Additionally, KEGG analysis identified enrichment in pathways such as chemical carcinogenesis—reactive oxygen species and steroid hormone biosynthesis, suggesting these genes may play roles in processes like xenobiotic metabolism and carcinogenesis. These analyses provide insights into the potential functions and pathways associated with our core genes, laying a foundation for further investigation into their roles in network toxicology (Figure 3A,B).

### 2.7. Immunoinfiltration and Drug Sensitivity Analysis

In the ssGSEA analysis, the *x*-axis represents different immune cell types, while the *y*-axis indicates the expression levels of genes within these immune cells. The analysis revealed that in CD4+ T cells, the proportion of AURKA was significantly higher in the high-expression group, whereas CYP1A2 and ESR1 were more prevalent in the low-expression group. Conversely, in B cells, CYP1A2 and ESR1 were predominantly found in the high-expression group, while AURKA was more common in the low-expression group.

The ESTIMATE analysis, which calculates the ESTIMATE score as the sum of the stromal score and immune score, demonstrated that a higher ESTIMATE score correlates with lower tumor purity. It was observed that the high-expression group of AURKA exhibited higher tumor purity, whereas the high-expression groups of CYP1A2 and ESR1 were associated with lower tumor purity.

**Figure 4:**
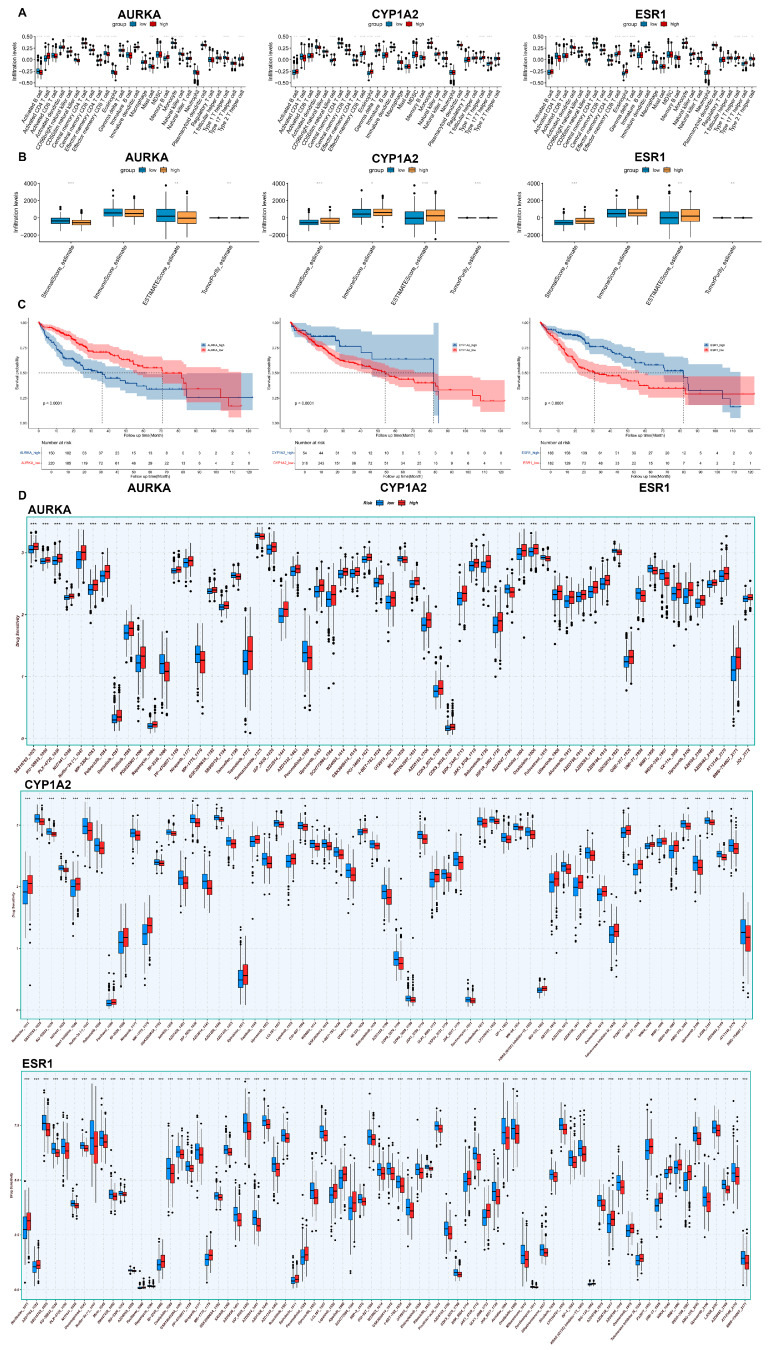
Multi-dimensional validation of core targets (AURKA, ESR1, CYP1A2). *** indicates *p* < 0.005. (**A**,**B**) Immune infiltration analysis assessing the correlation between target expression and immune cell abundance. (**C**) Survival analysis (Kaplan–Meier curves) evaluating the prognostic significance of core targets. (**D**) Drug sensitivity analysis linking target expression to therapeutic response.

Furthermore, a drug sensitivity analysis indicated that certain compounds, such as nutlin-3a, may exert potent inhibitory effects on CYP1A2, AURKA, and ESR1. These findings suggest that these genes play distinct roles in immune cell infiltration and tumor microenvironment composition, and they may serve as potential targets for therapeutic intervention. The differential expression patterns and drug sensitivity profiles of CYP1A2, AURKA, and ESR1 highlight their potential significance in the context of immune modulation and cancer therapy (Figure 4A,B,D).

### 2.8. Survival Analysis

In the study, we conducted a survival analysis to evaluate the prognostic significance of gene expression levels in patients with a specific disease. By plotting survival curves, we found that high or low expression levels of the ESR1, CYP1A2, and AURKA genes were significantly associated with patient survival time. Specifically, high expression of ESR1 and CYP1A2 was linked to improved survival outcomes, while low expression of AURKA was associated with worse prognoses. These findings suggest that these genes play critical roles in the progression of the disease and may serve as potential therapeutic targets. Furthermore, the strong correlation between gene expression levels and survival time highlights the importance of these genes as core determinants of disease outcomes. These results provide valuable insights into the molecular mechanisms underlying the disease and offer a foundation for further functional studies to validate their roles in prognosis and therapy (Figure 4C).

### 2.9. Molecular Docking

**Figure 5:**
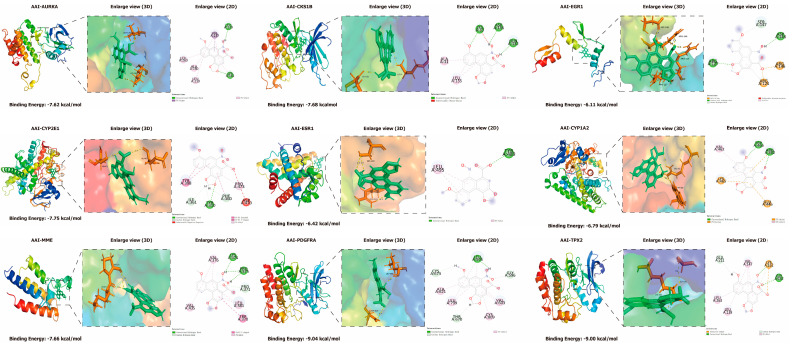
Molecular docking analysis of core targets and AAI.

Through molecular docking analysis, we identified that PDGFRA, TPX2, CYP2E1, CKS1B, MME, AURKA, CYP1A2, and ESR1 exhibited binding energies with AAI (aristolochic acid I) of less than −6 kcal/mol, further confirming the strong binding affinity of CYP1A2, AURKA, and ESR1 to AAI. An enlarged visualization of the docking results revealed that AAI interacts with several key amino acid residues in these proteins. Specifically, AAI formed hydrogen bonds with LEU-136 and LYS-162 of AURKA; LYS-33, ASP-146, and GLN-132 of CKS1B; TRP-466 and ARG-362 of CYP1A2; TYR-310 of CYP2E1; ASP-148 and ARG-127 of EGR1; TYR-489 of ESR1; LYS-374 and PRO-375 of MME; LEU-595 of PDGFRA; and GLY-142 of TPX2. These interactions highlight the potential binding sites of AAI, an environmental pollutant and carcinogen, which may contribute to its role in the development of HCC. These findings provide molecular-level insights into the mechanisms through which AAI exerts its toxic and carcinogenic effects, emphasizing the importance of these proteins as potential targets for further investigation and therapeutic intervention (Figure 5).

### 2.10. Molecular Dynamics Simulations

To further validate the binding affinity of AAI to the three core targets (ESR1, CYP1A2, and AURKA), we performed molecular dynamics (MD) simulations. Root Mean Square Deviation (RMSD) was used to assess the conformational stability of the protein-ligand complexes, reflecting the degree of the deviation of atomic positions from their initial configurations. Lower RMSD values indicate greater conformational stability. all AAI complexes reached equilibrium after 90 ns of simulation. Notably, the AAI-ESR1 and AAI-CYP1A2 complexes exhibited lower RMSD values (approximately 1.75 Å) compared to the AAI-AURKA complex, suggesting particularly stable interactions. In contrast, the AAI-AURKA complex showed slightly higher RMSD fluctuations, indicating relatively less stability.

Root Mean Square Fluctuation (RMSF) was employed to evaluate the flexibility of amino acid residues in the proteins. The RMSF values for the amino acid residues in the AAI-ESR1 and AAI-CYP1A2 complexes were generally lower (mostly between 1 and 6 Å), indicating reduced flexibility and increased binding stability. In contrast, the AAI-AURKA complex displayed higher RMSF peaks, suggesting greater flexibility in certain regions. Despite these differences, all three target proteins demonstrated stable binding with AAI throughout the simulation.

**Figure 6:**
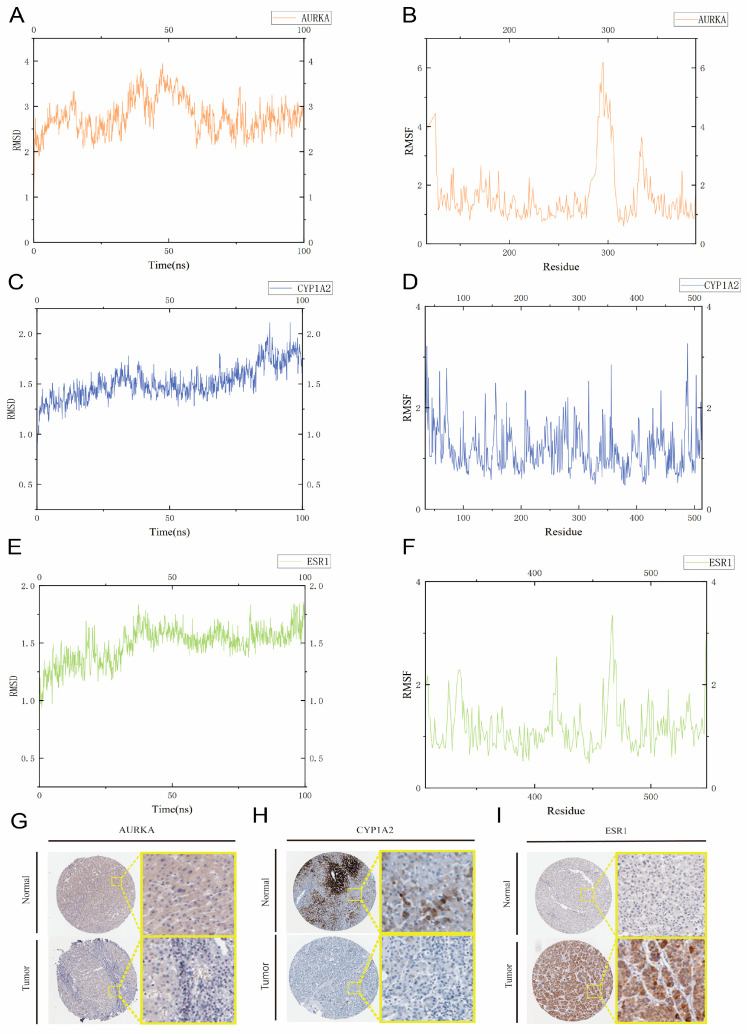
Molecular dynamics simulation and IHC. (**A**–**F**) Molecular dynamics simulation of AAI bound to AURKA, ESR1, and CYP1A2, evaluating complex stability and dynamic interactions over time. (**G**–**I**) IHC image for AURKA, CYP1A2, and ESR1 from the HPA database.

In summary, the MD simulation results confirm that AAI forms stable complexes with ESR1, CYP1A2, and AURKA, with AAI-ESR1 and AAI-CYP1A2 exhibiting particularly strong and stable interactions. These findings provide valuable insights into the molecular mechanisms underlying AAI’s binding affinity and its potential biological effects on these core targets (Figure 6A–F).

### 2.11. Immunohistochemistry Based on the HPA Database

We obtained liver tissue samples from the HPA database, including normal liver and HCC tissues. In normal tissues, ESR1 was negative; in HCC samples, ESR1 showed moderate staining. In normal tissues, AURKA exhibited weak positivity, whereas it was negative in HCC samples. CYP1A2 displayed high-intensity staining in normal tissues but only moderate intensity in HCC samples. These observations further corroborate the conclusions drawn from our study (Figure 6G–I).

## 3. Discussion

This study first utilized extensive bioinformatics data from databases such as PubChem, OMIM, and ChEMBL to identify potential targets associated with AAI-induced HCC [34]. Through machine learning, the STRING platform, Cytoscape software, and gene enrichment analysis, we identified key genes more strongly correlated with HCC development and explored their potential roles in cellular metabolism [35], steroid hormone synthesis, and oxidative stress responses, providing new insights into the carcinogenic mechanisms of AAI [36]. As computational models are increasingly used to determine toxicological pathways, comprehensively investigating the molecular mechanisms of harmful substances may aid in the discovery of valuable therapeutic targets [37,38]. In addition to elucidating the molecular mechanisms of AAI-induced HCC, this study also analyzed the impact of AAI exposure on the tumor microenvironment through immune microenvironment analysis, revealing that different gene expression levels are closely related to immune cell infiltration and tumor microenvironment composition [32], suggesting their potential significance in immune regulation and cancer therapy [39]. Furthermore, survival analysis indicated that the expression levels of CYP1A2, ESR1, and AURKA are significantly associated with the prognosis of liver cancer patients, providing potential biomarkers for clinical treatment [40,41]. These findings may broaden current perspectives on the carcinogenic mechanisms of AAI and could serve as a preliminary theoretical reference for future targeted and immunotherapy approaches [42]. This study systematically elucidates the potential mechanisms of AAI, an environmental pollutant, in inducing hepatocellular carcinoma, identifying key targets including CYP1A2, ESR1, and AURKA [43,44]. Given the persistent environmental presence of AAI and the high prevalence of HCC, our findings contribute to novel strategies and targets for the early diagnosis and treatment of hepatocellular carcinoma.

CYP1A2 is the primary enzyme responsible for the formation of toxic AFBO (the major metabolite of aflatoxin B1) globally [45]. The polymorphic variants of CYP enzymes influence the susceptibility to AFB1-induced HCC [46]. Variations in CYP1A2 and CYP3A4 affect the rate and extent of AFB1 bioactivation, thereby influencing an individual’s susceptibility to HCC [47]. Among HCC patients, the activity of CYP isoforms varies, with CYP3A4, CYP3A5, CYP3A7, CYP2B7, and CYP3A3 being involved in the bioactivation of mutagenic metabolites of AFB1 [48]. A significant variability in the frequency of CYP2D6*10* has been observed in HCC patients [49]. Both rs2740574 (an upstream polymorphism of CYP3A41B) and rs776746 (affecting CYP3A5 RNA splicing) influence the expression of CYP3A, thereby affecting the variability of AFB1-epoxide adducts in HCC patients [50]. Furthermore, studies indicate that CYP1A2 modulates the biochemical effects of the anti-angiogenic drug axitinib in the treatment of advanced HCC [51]. In addition, several studies have confirmed that CYP1A2 also possesses a certain metabolic capacity for AAI; by inducing the expression of CYP1A2, the formation of carcinogenic AAI–DNA adducts can be inhibited, thereby reducing the risk of cancer development [52]. A 2025 study further reported that stir-fried Semen Armeniacae Amarum may serve as a novel drug candidate for attenuating AAI-induced hepatotoxicity; its protective mechanism is likely linked to the modulation of transporters and metabolic enzymes [53].

In recent years, increasing evidence suggests that genetic variations in hormone-related genes are key carcinogenic factors [54]. ESR1, one of the genes encoding estrogen receptor alpha, is located on chromosome 6 at the 6p25.1 locus. ESR1 is a ligand-activated transcription factor composed of several domains crucial for DNA binding, hormone regulation, and transcriptional activation. Its epigenetic and genetic alterations may result in differential estrogen metabolism, while also interacting with estrogen receptors to stimulate changes in the expression of downstream genes and the proliferation of mammary epithelial tissue [55]. Numerous studies have shown a potential association between genetic variations in ESR1 and cancer risk, including rs1801132 (C > G) located in exon 4 and rs2077647 (A > G) in exon 1 (S10S) [56]. The role of ESR1 polymorphisms in carcinogenesis and progression is still under investigation, but it has been identified as a candidate gene for cancer susceptibility [57]. Polymorphisms in ESR1, such as PvuII (rs2234693 T > C), XbaI (rs9340799 A > G), and T594T (rs2228480 G > A), have been reported to be significantly associated with cancer development, particularly in HCC and prostate cancer, as confirmed by this study [58,59].

Aurora kinase A (AURKA) is a key protein that regulates cell growth and division. It plays a major role in cancer development and progression by inducing abnormal cell behaviors [60]. AURKA is often overexpressed in HCC, making it a potential diagnostic and prognostic marker for disease severity [61]. The emerging role of AURKA as a hub gene in HCC highlights its importance as a fundamental kinase and a potential therapeutic target. Its function extends beyond the phosphorylation of direct substrates, integrating into a complex regulatory network involving various levels of ncRNA. Specifically, AURKA can regulate miRNA expression and promote chemotherapy resistance in HCC by overexpressing the NF-κB/miR-21/PTEN/AKT signaling axis, emphasizing AURKA’s potential in modulating multiple signaling pathways [62]. Growing evidence is exploring the use of AURKA as a target for novel anticancer therapies in HCC [63].

GO and KEGG enrichment analyses suggest that several signaling pathways in HCC are related to the impact of AAI on HCC. Among these, tryptophan metabolism via the tryptophan–kynurenine–aromatic hydrocarbon receptor (Trp-Kyn-AhR) pathway is involved in physiological immune suppression and plays a role in acquired and intrinsic resistance to immunotherapy [64]. The tryptophan metabolic pathway has been shown to influence the development of non-alcoholic fatty liver disease (NAFLD), including non-alcoholic fatty liver, non-alcoholic steatohepatitis, cirrhosis, and even HCC [65]. Indole, a major gut bacterial product derived from tryptophan, includes indole-3-acetic acid (I3A), indolepropionic acid (IPA), indole-3-lactic acid, indole-3-carboxylic acid, and tryptamine [66]. Both I3A and tryptamine reduce macrophage production of pro-inflammatory cytokines and inhibit macrophage migration toward monocyte chemoattractant protein-1. Furthermore, I3A alleviates cytokine-mediated hepatocyte lipogenesis by activating the aryl hydrocarbon receptor [67]. This study suggests that I3A and tryptamine are key metabolites mediating host–microbiota crosstalk [68].

Among these, the one-carbon metabolism supports the degradation of tryptophan within cells [69]. In studies of liver diseases and fibrosis, several pathways within the one-carbon metabolism play significant roles in regulating energy metabolism and immune functions but have received limited attention [49]. Future research will offer more opportunities for exploration in this area [70].

Bile acid metabolism is tightly regulated by bile acid synthesis in the liver and bile acid biotransformation in the intestine [71]. Bile acids (BAs) are endogenous ligands that activate a complex network of nuclear receptor farnesoid X receptor (FXR) and membrane G-protein-coupled bile acid receptor-1 (TGR5) [72]. Disruptions in BA homeostasis lead to cholestatic liver diseases, gastrointestinal inflammatory disorders, obesity, and diabetes, making them potential therapeutic targets for metabolic disorder drugs [73]. Recent observations highlight that BAs function as hormonal regulators of cholesterol, energy, and glucose homeostasis by binding to specific receptors [74]. The nuclear hormone receptor FXR and the membrane receptor TGR5, are among the most studied. FXR is primarily expressed in the liver, intestine, kidney, and adrenal glands [75]. FXRα controls BA synthesis, transport, and detoxification. The activation of FXRs by BAs decreases the expression of key enzymes involved in BA biosynthesis, such as Cyp7a1 and Cyp8b1 [76]. In the liver, FXRα induces the transcription of target genes encoding small heterodimer partners (SHP, NR5O2), which act as transcriptional repressors [77]. Moreover, FXRα plays a key regulatory role in BA transport by influencing BA transporter expression [78]. In addition, the BA membrane receptor TGR5 prevents liver inflammation and carcinogenesis by inhibiting the macrophage NF-κB axis [79]. Beyond traditional BA receptors, the pregnane X receptor (PXR), vitamin D receptor (VDR), and constitutive androstane receptor (CAR) may also play roles in NAFLD progression and HCC [80]. Other genetic pathways, including steroid hormone biosynthesis, chemical carcinogen-receptor activation, chemical carcinogen–DNA adduct formation, cytochrome P450 metabolism of exogenous substances, and endocrine signaling, also influence the occurrence of HCC [81].

Moreover, additional studies have indicated that the carcinogenicity of aristolochic acid is chiefly ascribed to its reactive N-sulfated metabolite, N-sulfonyloxy-aristolactam (N-OSO_3_^−^-AL), which generates stable DNA–aristolactam adducts through a free-radical mechanism [82]. This suggests that the covalent binding of aristolochic acid to DNA may constitute a critical mechanism underlying its carcinogenic activity, and further investigations are warranted to elucidate the precise molecular pathways involved.

Although this study thoroughly investigates the potential molecular mechanisms of AAI-induced HCC, it completely relies on computational modeling techniques to infer the relationship between AAI and HCC [83]. In vivo experimental validation is lacking. For instance, the role of AAI with different exposure doses and at different times in the onset of HCC still requires further confirmation through animal experiments and patient sample analyses [84]. Further in vitro and in vivo validation will still be required in the future to strengthen the reliability of these findings.

Nevertheless, this study has built a systems biology model for AAI-induced carcinogenesis, systematically screening potential core targets of AAI-induced HCC through a network toxicology analysis. Additionally, using machine learning algorithms and SHAP analysis, 11 core genes were further selected, and their importance in machine learning was visualized, providing precise molecular targets for HCC mechanism research. Subsequently, an AAI-related PPI (protein–protein interaction) network was constructed and analyzed using the CytoHubba and MCODE algorithms in Cytoscape, identifying key genes. GO and KEGG enrichment analyses were performed to explore potential pathways of these genes [85]. Based on these analyses, ESR1, CYP1A2, and AURKA were identified as key genes, and their significant roles in HCC progression and prognosis were validated through immune infiltration, drug sensitivity analysis, and survival curves [86]. Finally, molecular docking and molecular dynamics simulations confirmed stable binding between AAI and CYP1A2, ESR1, and AURKA, providing important scientific evidence for the development of new drugs and targeted therapeutic strategies. Future studies should further elucidate the binding modes between AAI and these three key targets (ESR1, CYP1A2, and AURKA) and their potential impacts on oncogenic pathways, with the goal of developing targeted therapeutic agents that effectively inhibit AAI’s carcinogenic properties.

## 4. Conclusions

Our study integrates network toxicology, machine learning, molecular docking, and dynamics simulations to elucidate the molecular mechanisms of AAI-induced HCC. We identified CYP1A2, ESR1, and AURKA as key hub genes, demonstrating their critical roles in AAI toxicity and HCC progression. Molecular docking and dynamics simulations confirmed there were stable interactions between AAI and these targets, highlighting their potential as therapeutic intervention points. Immune infiltration and drug sensitivity analyses further revealed their influence on the tumor microenvironment and response to anticancer drugs. These findings allow for a comprehensive understanding of AAI’s carcinogenic mechanisms and offer valuable insights for the development of targeted strategies to mitigate its health risks.

## 5. Methods and Materials

### 5.1. Study Design

The design of this study is illustrated in the graphical abstract. This study was designed to investigate the molecular mechanisms of AAI-induced carcinogenicity, with a particular focus on its role in HCC. To reach its goals, this study proceeded in four stages. First, network toxicology and machine learning were used to identify critical targets. Second, molecular docking assessed how AAI binds to these targets. Then, molecular dynamics simulations confirmed the stability and dynamic behavior of the resulting complexes. Our study was validated by IHC from the HPA database (Graphical Abstract).

### 5.2. Forecasting Toxicity Effects of AAI

To comprehensively identify the molecular targets associated with AAI, we first retrieved its chemical structure and canonical SMILES representation through the PubChem database [87] using the obtained SMILES notation. We began by retrieving the SMILES notation for AAI from the PubMed database, which served as the foundational input for our toxicity prediction models. Our evaluation included comprehensive insights from the ADMET “https://admetmesh.scbdd.com/ (accessed on 2025/1/28)” and ProTox3 “https://tox.charite.de/protox3/ (accessed on 2025/1/28)” databases. The ADMET database provides valuable information regarding the pharmacokinetic properties and potential toxicity endpoints of AAI, whereas ProTox3 facilitates detailed predictions of various toxicity outcomes, such as hepatotoxicity, carcinogenicity, and mutagenicity. By integrating data from these resources, we developed a robust toxicity profile for AAI, identifying critical toxicological pathways and the potential health risks associated with AAI exposure.

### 5.3. Search for AAI Targets

To identify human-relevant protein interactions for AAI, we initiated our search in the ChEMBL database “https://www.ebi.ac.uk/chembl/ (accessed on 28 January 2025)”, specifically filtering for “*Homo sapiens*” as the target species. This approach ensured that only protein interactions pertinent to humans were included. We subsequently extended our investigation by inputting the SMILES notation of AAI into two additional databases: STITCH “http://stitch.embl.de/ (accessed on 28 January 2025)” and SwissTargetPrediction “http://www.swisstargetprediction.ch/ (accessed on 28 January 2025)”. To maintain data precision, we carefully cross-validated the protein targets obtained from all three databases and standardized them using the UniProt database. Through this meticulous process, we compiled a curated and extensive list of 962 potential AAI targets. This dataset formed the basis for subsequent analyses aimed at elucidating the interactions between AAI and critical proteins implicated in HCC.

### 5.4. Identification of Targets Associated with HCC

To identify potential molecular targets associated with HCC, we utilized three reputable bioinformatics databases: OMIM “https://omim.org/ (accessed on 28 January 2025)”, TTD “https://db.idrblab.net/ttd/ (accessed on 28 January 2025)”, and GeneCards “https://www.genecards.org/ (accessed on 28 January 2025)”. To ensure the significance of our findings, we established a strict criterion by applying a “GeneCards Inferred Functionality Score” (GIFtS) threshold, specifically including only those genes with a GIFtS value greater than 10. This criterion aimed to guarantee a strong association between the genes and the disease. In total, we identified 1888 genes that were strongly associated with disease.

### 5.5. Recognition of Core Genes from GEO

To validate and identify core genes associated with the toxicity of AAI, we integrated three gene expression datasets from the GEO database, i.e., GSE14520, GSE36376, and GSE76427 in R. The datasets were merged using the “ComBat” package, which effectively adjusted for batch effects to ensure data consistency. PCA was then performed to visualize the distribution and clustering of the samples, confirming the successful integration of the datasets. We subsequently applied the “limma” package in R to identify DEGs, resulting in the identification of 417 significant DEGs. To highlight the most prominent changes, the top ten upregulated and downregulated DEGs were visualized using a volcano plot. Additionally, we intersected the DEGs from the three datasets, yielding 18 core genes with consistent expression patterns across all datasets. These genes were further visualized via multigroup boxplots to illustrate their expression profiles in tumor and tumor-adjacent samples. This comprehensive approach provided robust insights into the molecular mechanisms underlying AAI toxicity and identified key genes for further functional validation.

### 5.6. Machine Learning Powered Genetic Assessment 

To validate and identify core genes associated with the toxicity of AAI, we employed three machine learning algorithms: SVM-RFE, random forest, and LASSO regression. These methods were applied to screen and prioritize genes on the basis of their importance and relevance to AAI-induced toxicity. SVM-RFE was used to iteratively eliminate less significant features, whereas random forest was used to evaluate gene importance through ensemble learning. LASSO regression further refined the selection by penalizing less relevant features, ensuring a robust set of candidate genes. To validate the reliability of the selected genes, we constructed ROC curves using ten additional machine learning algorithms, which demonstrated strong predictive performance. Finally, we performed SHAP analysis to interpret the contribution of each gene to the model’s predictions, identifying the most influential genes driving the toxicity mechanisms. This comprehensive approach ensured the identification of high-confidence core genes for further functional validation and mechanistic studies.

### 5.7. Construction of Protein–Protein Interaction Network

To explore the molecular mechanisms underlying AAI-induced HCC, we identified common potential targets between AAI and HCC by analyzing their intersection, which represents potential targets associated with AAI-induced HCC. To investigate the interactions among these targets and identify critical proteins involved in HCC pathways, we constructed a PPI network using a multi-step approach. First, the common targets were submitted to the STRING database “http://string-db.org (accessed on 28 January 2025)”, a widely used platform for predicting PPIs on the basis of experimental data, computational predictions, and text mining. To ensure human-specific relevance, we restricted the analysis to *Homo sapiens* and applied a high-confidence interaction threshold (score > 0.4), ensuring the inclusion of only the most reliable interactions. The resulting interaction data were exported and then imported into Cytoscape software (version 3.10.2) for network visualization and analysis.

### 5.8. Hub Target Screening

To confirm and identify the key genes in the network, we used the CytoHubba and MCODE tools in Cytoscape software. CytoHubba was used to study the role and importance of genes in the network using twelve different methods: betweenness, bottle neck, closeness, clustering coefficient, degree, DMNC, eccentricity, EPC, MCC, MNC, radiality, and stress. We applied these methods to the key genes we obtained from the STRING database. This allowed us to fully understand the position and role of each gene in the network. The betweenness method revealed genes that act as bridges between other genes. The degree method highlighted the genes with the most connections. The closeness and radiality methods focused on the genes that were closest to others in the network. The stress and eccentricity methods looked at genes that were important for keeping the network stable. We also used the MCODE tool to find tightly connected groups of genes and to identify important smaller networks and key genes. By combining these approaches, we were able to find the most important genes and smaller networks. These findings provide a strong basis for further study of gene functions and pathways in our network toxicology research.

### 5.9. Functional Enrichment Profiling via GO and KEGG

We performed GO and KEGG enrichment analyses using 11 core genes to elucidate their potential roles in biological processes. The GO analysis results were divided into three categories: molecular function (MF), biological process (BP), and cellular component (CC).

### 5.10. Immuneinfiltration and Drug Sensitivity Analyses

To explore the functional implications of the 11 core genes, we conducted immune infiltration and drug sensitivity analyses. Specifically, we focused on AURKA, ESR1, and CYP1A2, dividing their expression levels into upregulated and downregulated groups on the basis of the top 50% and bottom 50% thresholds. Using the ssGSEA algorithm, we evaluated the distribution of immune cell populations in both groups, revealing differences in immune infiltration patterns. Additionally, the ESTIMATE algorithm was applied to assess the tumor microenvironment (TME) scores, including the stromal and immune scores, for the upregulated and downregulated groups. The findings provided insights into the relationships between gene expression and TME characteristics. Furthermore, drug sensitivity analysis was performed using the R4.3.3 package “oncoPredict”, which predicted the response of both groups to various anticancer drugs. The results highlighted distinct drug sensitivity profiles between the upregulated and downregulated groups, suggesting potential therapeutic targets. Together, these analyses deepened our understanding of the roles of these core genes in immune regulation and drug response within the context of network toxicology.

### 5.11. Survival Analysis Using Samples from the TCGA Database

To evaluate the clinical relevance of ESR1, CYP1A2, and AURKA in cancer, we utilized RNA-seq data from tumor samples in The Cancer Genome Atlas (TCGA) database. For each gene, we stratified the samples into high-expression and low-expression groups on basis of the median expression level. A Kaplan—Meier survival analysis was then performed to compare overall survival (OS) between the two groups. The log-rank test was applied to assess the statistical significance of the differences in survival outcomes. Survival curves were plotted to visualize the relationship between gene expression levels and patient survival. This approach allowed us to determine whether the high or low expression of ESR1, CYP1A2, and AURKA was associated with better or worse prognosis in cancer patients. The results provided insights into the potential roles of these genes as biomarkers for cancer progression and survival.

### 5.12. Molecular Docking of AAI and Its Core Targets

To investigate how AAI interacts with the core target proteins identified in our network analysis, we obtained the crystal structures of the core proteins from the RCSB Protein Data Bank (PDB), ensuring their accuracy and experimental validation. Prior to docking, we prepared the protein structures using PyMOL 1.8.6 by eliminating any existing ligands and water molecules. This preparation was crucial for establishing an unobstructed binding site for AAI. The proteins were subsequently processed to remove water molecules and hydrogen atoms were added. We defined a grid box around the active site of each protein to concentrate the docking analysis on the most pertinent binding regions. For each protein–ligand pair, we utilized the “dock_run.mcr” script in YASARA for molecular docking, and the docking model exhibiting the lowest binding energy was selected as the definitive result. We employed PyMOL and Discovery Studio 2024 to visualize the binding interactions, emphasizing critical details such as hydrogen bonds and hydrophobic contacts. This visualization provided valuable insights into the potential impact of AAI on these core proteins.

### 5.13. Molecular Dynamics Simulations for Core Genes

On the basis of the docking data and previous results, we selected complexes of AURKA, ESR1, and CYP1A2 for molecular dynamics simulations using the YASARA-structure tool. We used the AMBER14 force field to set up the simulation environment, which included a periodic boundary of 20 Å and water as the solvent, with a density of 0.998 g/mL. To ensure stability, we optimized the hydrogen bond network and adjusted the protonation state of the protein at pH 7.4 using pKa prediction. We neutralized the system by adding sodium chloride ions and minimized its energy using the YASARA-structure tool. Before running the 100 ns simulation, we resolved any clashes using simulated annealing and steepest descent methods. The simulation used the AM1BCC and GAFF2 force fields for oleuropein, TIP3P for water, and AMBER14 for the protein. We applied an 8 Å cutoff for van der Waals forces and no cutoff for electrostatic forces, using the Particle Mesh Ewald algorithm. The NPT ensemble was used to equilibrate the system, maintaining a temperature of 298 K and a pressure of 1 bar. We restrained positions to balance nonbonded and bonded interactions with multiple time steps of 2.5 and 5.0 fs. A Berendsen thermostat controlled the temperature and pressure, whereas a modified LINCS algorithm constrained the bond angles. The simulation was completed using ‘md_run.mcr’ macro in YASARA, and trajectory analysis was performed with ‘md_analyze.mcr’ and ‘md_analyzeres.mcr’. We took 400 snapshots at intervals of 250 ps to analyze and visualize the results using the YASARA-structure tool.

### 5.14. Immunohistochemistry Validation Form HPA Database

In order to further investigate the expression patterns of three key genes (AURKA, ESR1, and CYP 2) in normal and HCC tissues, the present study retrieved key gene expression data from the HPA database “https://www.proteinatlas.org/ (accessed on 23 July 2025)” for both normal liver and HCC tissues. This methodological approach served to enhance the reliability and accuracy of the research findings.

## Data Availability

The original contributions presented in this study are included in the article. Further inquiries can be directed to the corresponding author(s).

## Abbreviations

AAI: aristolochic acid I; HCC: hepatocellular carcinoma; SBS22: single-base substitution mutational signature 22; AAN: aristolochic acid nephropathy; ADMET: absorption, distribution, metabolism, excretion, and toxicity; ROC: receiver operating characteristic; AUC: area under the curve; SHAP: SHapley additive exPlanations; PPI: protein–protein interaction; GEO: gene expression omnibus; DEGs: differentially expressed genes; GIFtS: GeneCards-inferred functionality score; SVM-RFE: support vector machine-recursive feature elimination; PCA: principal component analysis; TCGA: The Cancer Genome Atlas; OS: overall survival; ssGSEA: single-sample gene set enrichment analysis; ESTIMATE: estimation of stromal and immune cells in malignant tumor tissues using expression data; TME: tumor microenvironment; PDB: protein data bank; RMSD: root mean square deviation; RMSF: root mean square fluctuation; AMBER: assisted model building with energy refinement; GO: gene ontology; KEGG: Kyoto Encyclopedia of Genes and Genomes; MF: molecular function; BP: biological process; CC: cellular component.
